# *MET* Mutation Is a Potential Therapeutic Target for Advanced Endometrial Cancer

**DOI:** 10.3390/cancers13164231

**Published:** 2021-08-23

**Authors:** Yu-Min Yeh, Pei-Ying Wu, Peng-Chan Lin, Pei-Fang Su, Ya-Ting Hsu, Keng-Fu Hsu, Meng-Ru Shen

**Affiliations:** 1 Department of Internal Medicine, National Cheng Kung University Hospital, College of Medicine, National Cheng Kung University, Tainan 704, Taiwan; i5485111@gmail.com (Y.-M.Y.); pengchanlin@gmail.com (P.-C.L.); yatinhsu@hotmail.com (Y.-T.H.); 2 Department of Obstetrics and Gynecology, National Cheng Kung University Hospital, College of Medicine, National Cheng Kung University, Tainan 704, Taiwan; anna1002ster@gmail.com; 3 Department of Computer Science and Information Engineering, College of Electrical Engineering and Computer Science, National Cheng Kung University, Tainan 704, Taiwan; 4 Department of Statistics, College of Management, National Cheng Kung University, Tainan 704, Taiwan; pfsu@ncku.edu.tw; 5 Graduate Institute of Clinical Medicine, College of Medicine, National Cheng Kung University, Tainan 704, Taiwan; 6 Department of Pharmacology, College of Medicine, National Cheng Kung University, Tainan 704, Taiwan

**Keywords:** endometrial cancer, *MET* mutation, germline variant, targeted therapy, chemoresistance

## Abstract

**Simple Summary:**

Endometrial cancer is the most common gynecological cancer in developed countries. At initial diagnosis, extra-uterine spread is observed in about 25% of patients. Chemotherapy is the suggested mode of treatment for patients with extra-uterine metastasis; however, the 5-year overall survival rate of these patients remains poor. The development of new therapeutic strategies to improve the poor clinical outcome of patients with advanced endometrial cancer is still in great demand. In this study, we aim to understand the genomic landscape of advanced endometrial cancer and identify new therapeutic targets. Integrated genomic, pathological, and clinical data are analyzed to identify the survival-associated genomic alterations. In addition, the impacts of the genomic alterations are examined in silico, in vitro, and in vivo. The results of this study may aid in developing biomarker-guided treatments for patients with advanced endometrial cancer.

**Abstract:**

An optimal therapeutic regimen for endometrial cancer with extra-uterine metastasis is unavailable. This study aims to improve our understanding of the genomic landscape of advanced endometrial cancer and identify potential therapeutic targets. The clinical and genomic profiles of 81 patients with stage III or IV endometrial cancer were integrated. To identify genomic aberrations associated with clinical outcomes, Cox proportional hazard regression was used. The impacts of the genomic aberrations were validated in vitro and in vivo. The mutation status of *MET*, *U2AF1*, *BCL9*, *PPP2R1A*, *IDH2*, *CBL*, *BTK,* and *CHEK2* were positively correlated with poor clinical outcomes. *MET* mutations occurred in 30% of the patients who presented with poor overall survival (hazard ratio, 2.606; 95% confidence interval, 1.167~5.819; adjusted *p*-value, 0.067). Concurrent *MET* and *KRAS* mutations presented with the worst outcomes. *MET* mutations in hepatocyte growth factor (HGF)-binding (58.1%) or kinase (16.2%) domains resulted in differential HGF-induced c-MET phosphorylation. Different types of *MET* mutations differentially affected tumor growth and displayed different sensitivities to cisplatin and tyrosine kinase inhibitors. *MET* N375S mutation is a germline variant that causes chemoresistance to cisplatin, with a high incidence in Eastern Asia. This study highlights the ethnic differences in the biology of the disease, which can influence treatment recommendations and the genome-guided clinical trials of advanced endometrial cancer.

## 1. Introduction

Endometrial cancer, the most common gynecological cancer in developed countries, is broadly classified into two types (type I and type II) based on its histology and the presence or absence of a hormone receptor [1]. Type I endometrioid cancer is associated with estrogen excess, obesity, hormone-receptor positivity, and a favorable prognosis, as compared to type II endometrial cancers, which present as serous tumors that are more common in non-obese women and exhibit a poorer outcome [2]. The 5-year overall survival rate ranges from 74% to 91% at the early stage (International Federation of Gynecology and Obstetrics (FIGO) stages I or II). In contrast, the 5-year overall survival rates in patients with extra-uterine metastatic disease are 57–66% and 20–26% for FIGO stages III and IV, respectively [3]. Extra-uterine spread is observed in about 25% of patients who are newly diagnosed with endometrial cancer, and chemotherapy is the suggested mode of treatment. However, the optimal treatment of this cancer has not been determined so far. In general, platinum-based chemotherapy is used as the first-line treatment for metastatic or advanced endometrial cancer. However, no standard protocol for the second-line option exists when the tumor persists or recurs [4].

The Cancer Genome Atlas (TCGA) Research Network has produced a vast amount of data on the genomic landscape of endometrial cancer [5]. The genomic analysis categorized endometrial cancer into subgroups based on distinct molecular characteristics: the group with DNA polymerase epsilon (*POLE*) somatic mutations and the corresponding ‘ultramutated’ phenotype exhibit a favorable prognosis, while the other distinct subgroups, which include tumors with microsatellite instability, low copy number, and high copy number, consist mainly of cases diagnosed as high-grade serous tumors with poor outcome. Systemic chemotherapy is the main treatment for patients with metastatic disease. Carboplatin plus paclitaxel is the preferred regimen for the majority of patients [4]. Hormone therapy could be used in selected patients with the expression of estrogen receptors and progesterone receptors [6], and immunotherapy targeting the programmed cell death 1 and programmed cell death ligand 1 interaction is approved for patients with mismatch repair deficient or high microsatellite instability endometrial cancer [7,8]. However, no targeted therapy for patients with specific predictive biomarkers has been approved so far. Therefore, the identification of the biological pathways that may be altered in advanced endometrial cancer might aid in improving the clinical management of this disease. 

This study aims to identify the survival-associated molecular pathways in advanced endometrial cancer via integrated genomic characterization. 

## 2. Materials and Methods

Study cohort. Patients with endometrial cancer were consecutively recruited at the National Cheng Kung University Hospital Taiwan from July 2006 to January 2017. About 900 patients with endometrial cancer were enrolled, and 20% of cases were diagnosed with advanced-stage cancer (FIGO stage III or IV). The patients received staging surgery or diagnostic dilation and curettage at initial diagnosis. Platinum plus paclitaxel were the standard regimens used as postoperative chemotherapy for patients with FIGO stage III and IV. The patients were recruited as an NCKUH cohort using the following inclusion criteria: (i) FIGO stage III or IV; (ii) surgery performed at NCKUH; and (iii) adequate and qualified specimens for genetic analysis. The exclusion criteria were as follows: (i) unavailable clinical information; (ii) death unrelated to cancer; and (iii) poor quality of specimens. This study was approved by the institutional review board of NCKUH (A-ER-103-151, A-ER-103-395, and A-ER-104-153). Whole-genome sequencing (WGS) data of 499 normal Taiwanese individuals were provided by the Taiwan Biobank as a genome reference.

Next-generation sequencing. Genomic analysis was performed on formalin-fixed paraffin-embedded (FFPE) tumor tissues using Oncomine Comprehensive Assay™ v1 (ThermoFisher, Waltham, MA, USA) [9]. Hematoxylin and eosin (H&E)-stained FFPE sections were reviewed to ensure that the tumor content was >50%. Genomic DNA and RNA were extracted from the sections using the Recover All Total Nucleic Acid Isolation Kit (Thermo Fisher Scientific). RNA was reverse-transcribed to produce cDNA using the SuperScript VILO cDNA Synthesis Kit (Thermo Fisher Scientific). Target regions from genomic DNA and cDNA were amplified using DNA Oncomine Cancer Research and RNA Oncomine Cancer Research panels (Thermo Fisher Scientific). Library construction of the amplicons was performed according to the manufacturer’s instructions using the Ion AmpliSeq Library Kit 2.0 (Thermo Fisher Scientific). The template was prepared with the Ion PGM Template OT2 200 Kit (Thermo Fisher Scientific), and the Ion 318 chip (Thermo Fisher Scientific) was prepared and loaded according to the manufacturer’s recommendations. The Ion PGM Sequencing 200 Kit v.2 (Thermo Fisher Scientific) was used with the Ion PGM sequencer (Thermo Fisher Scientific), as described in the User Guide (average sequencing depth, 1000×).

Next, *POLE* gene panels were designed and ordered at AmpliSeq.com to identify the pathogenic mutations in the gene. *POLE* gene libraries were prepared using the Ion AmpliSeq™ Kit for Chef DL8 with the Ion Chef™ System. Template preparation, chip loading, and sequencing were carried out on the Ion Chef™ System and the Ion S5 XL sequencing system using the Ion 510 & Ion 520 & Ion 530 Kit-Chef Kit (Thermo Fisher Scientific). The sequencing reads were aligned to the reference genome (hg19), and variant calling and annotation were conducted using Ion Reporter Version 5.6. 

To detect the germline genetic variant, DNA was isolated from the peripheral blood of 13 patients and adjacent non-tumor specimens of 25 patients. WGS and whole exome sequencing (WES) of the blood and tissue samples were performed using the Illumina HiSeq^®^ 2500 and Ion Torren^TM^ systems, respectively. NGS library preparation was carried out using the TruSeq PCR-Free DNA HT Library Prep Kit (Illumina Inc., San Diego, CA, USA). The size and concentration of the DNA library were measured using an Agilent 2100 bioanalyzer (Agilent, Santa Clara, CA, USA) and Qubit fluorometer (ThermoFisher, Waltham, MA, USA). WGS and WES were performed with a minimum median coverage of 30× and 100×, respectively. FastQC was used to check the quality of the reads, which were aligned to the hg19 reference genome using the BWA-MEM algorithm. GATK Best Practices was used for base quality score recalibration, small insertion and deletion (INDEL) realignment, and duplicate removal [10]. Single nucleotide variant (SNV) and INDEL discovery and genotyping were performed according to GATK [11,12]. Manta and Canvas were used for SNV and CNV discovery [13,14]. To study the possible functional impacts of the associated variants, the Ensemble Variant Effect Predictor was used [15]. The sequencing coverage and quality statistics for each sample are summarized in Table S1.

Site-directed mutagenesis of *MET*. The pCMV6-*MET* mutant (N375S) plasmid was purchased from ORIGENE (#RC400336). The construction of mutated derivatives of *c-MET*, including the G1085R and G1087E, was performed using the QuikChange site-directed mutagenesis kit (Agilent Technologies). Mutations for target nucleotide were introduced using the designed oligonucleotides and pCMV6-*MET* (ORIGENE; #RC217003) as a template. The PCR products were added to the DpnI enzyme for 5 min at 37 °C to destroy the parental plasmid DNA and then transformed into *E. coli*. All mutated constructs were confirmed by sequencing.

Cell culture and functional assays. In April 2017, authenticated endometrial cancer cell lines, RL95-2 (RRID: CVCL_0505) and KLE (RRID: CVCL_1329), were purchased from the American Type Culture Collection. All cell lines have been authenticated using short tandem repeat profiling within the last three years. The RL95-2 and KLE cell lines were transfected with wild-type and mutant *MET*, including N375S, G1085R, and G1087E, for functional assays. Cell functions on proliferation, migration, and invasion were performed as previously described. Briefly, the RL95-2 and KLE cell lines, transfected with wild-type and mutant *MET,* were seeded into 96-well plates (5 × 10^3^/well) with 100 μL of culture medium and incubated at 37 °C with 5% CO_2_. Cell proliferation was evaluated at 0, 24, 48, and 72 h after seeding using the Alamar Blue assay (Invitrogen). Next, a transwell assay was used to evaluate the migration and invasion abilities of the transfected cells. For the invasion assay, 100 μL of matrigel (10%; BD Biosciences) was added to the bottom of the transwell insert. Next, 3 × 10^4^ transfected cells were seeded onto the transwell insert along with 300 μL of medium containing 5% FBS; 1 mL of medium with 10% FBS was added to the lower part of the 24-well plate. The cells were allowed to invade or migrate for 24 h at 37 °C with 5% CO_2_. All the cells from the underside of the transwell inserts were stained by crystal violet. Images of each transwell insert were taken to count the number of cells. All experiments were performed with mycoplasma-free cells.

c-MET phosphorylation. The cells were washed with phosphate-buffered saline (PBS) and lysed in cell lysis buffer (10 mM Tris pH7.4, 150 mM NaCl, 1 mM ethylenediaminetetraacetic acid [EDTA], 1% octylphenoxypolyethoxyethanol (IGEPAL), 0.5% deoxycholic acid, and 0.1% SDS) containing freshly added Protease Inhibitor Cocktail (Sigma, St. Louis, MO, USA) and 1 mM phenylmethanesulfonylfluoride (PMSF). The protein concentration of the cell lysate was determined using the DC Protein Assay (Bio-Rad Laboratories, Hercules, CA, USA). Equal amounts of lysate protein (30 µg) from each cell lysate were loaded onto 4–15% SDS-polyacrylamide gel (Bio-Rad) and separated by electrophoresis. The separated proteins were electroblotted onto a nitrocellulose membrane (0.45 µm; Bio-Rad) and incubated in blocking solution (1X PBS, 0.1% Tween-20, 5% non-fat dry milk powder) for 1 h at room temperature. The membranes were incubated with the following dilutions of the primary antibody at 40 °C overnight: anti-phospho-c-MET, 1:1000 (#3077; Cell Signaling Technology); anti-c-MET, 1:2000 (#8198; Cell Signaling Technology); and anti-α-tubulin, 1:5000 (DM1A; Novus Biologicals). After multiple washes with PBS containing 0.1% Tween-20 (PBST), the membranes were incubated with horseradish peroxidase-conjugated secondary antibody (goat anti-mouse or goat anti-rabbit IgG; Bio-Rad) for 1 h at room temperature. After further washes with PBST, the membranes were processed using the enhanced chemiluminescence method (SuperSignal West Pico substrate; Pierce, Rockford, IL, USA). Protein bands were visualized by autoradiography, and the signal intensities were quantified by using NIH ImageJ software.

Immunohistochemical staining. FFPE endometrial cancer tissues from surgical specimens were used to determine the expression of c-MET protein. Tissue sections were cut from the paraffin block (thickness, 4 μm), deparaffinized in xylene, and rehydrated with decreasing grades of ethanol. Immunohistochemical staining for the c-MET protein was performed using the anti-c-MET rabbit polyclonal antibody (sc-161; Santa Cruz Biotechnology). The expression level of c-MET in tumors with wild-type *MET* was compared with those in tumors carrying various *MET* mutations.

In silico analysis. To predict the effect of variants on the function of c-MET protein, differences in free energy between mutant and wild-type c-MET were calculated using Calculate Mutation Energy [16]. For the variants located in the tyrosine kinase domain, the analysis was performed using the crystal structure of the kinase domain in complex with ATP. Regarding the variants located in the Sema domain, the binding with hepatocyte growth factor (HGF) was used for estimation. Variants with mutation energy higher than 0.5 kcal/mol were reported as destabilized. 

Animal Study. All animal experiments were approved by the Laboratory Animal Care and Use Committee of the NCKU. The xenograft procedures were performed on 6–8-week-old, female, severe combined immunodeficient (NOD.CB18-Prkdc^scid^/JNarl, NOD/SCID) mice. The posterior flank of the NOD-SCID mouse was subcutaneously inoculated with 1 × 10^6^ endometrial cancer RL95-2 cells, expressing either wild-type or mutant *MET* (N375S, G1085R, G1087E). The mice were randomly divided into four groups (at least four mice in each group) and treated with cisplatin, crizotinib (a multiple tyrosine kinase inhibitor), SU11274 (a specific c-MET inhibitor), or none of the aforementioned drugs; the treatments were initiated 1 week after tumor inoculation. Cisplatin (1 mg/kg) and SU11274 (6 mg/kg) were administered twice a week via intraperitoneal (i.p.) injection for 5 weeks. Crizotinib (25 mg/kg), prepared with 0.5% hydroxypropylmethylcellulose, was administered by oral gavage twice a week for 5 weeks. Both tumor size and body weight were measured twice a week, and tumor volume was determined via caliper measurements of the tumor length (L) and width (W) according to the following formula: LW^2^/2. The mice were sacrificed at day 40, and the tumors were excised and weighed. 

Statistics. Chi-square tests, Fisher’s exact test, and unpaired *t*-tests were used to compare the differences between the groups. After adjusting for the stage of the tumor, the association between individual mutated genes and survival outcome was assessed using the Cox proportional hazards regression model. The correction for multiple comparisons was performed using the false discovery rate (FDR) method. The cutoff for the FDR adjusted *p-*value was 0.1. Progression-free survival (PFS) and overall survival (OS) were calculated from the date of surgery to recurrence and death, respectively. Kaplan–Meier curves and log-rank tests were used to estimate the survival functions and compare the differences between groups. A *p*-value of <0.05 was considered statistically significant.

## 3. Results

### 3.1. Patient Characteristics

Eighty-one patients with stage III and IV cancer were consecutively recruited based on the inclusion criteria. Based on the histology, 80% and 20% of the patients presented with type I (mainly endometrioid) and type II (non-endometrioid) tumors, respectively. The patients were grouped based on the clinical outcome, as follows: no evidence of disease and progressive disease (PD). The clinical characteristics of the patients in the two groups are shown in Table S2. No significant differences in histology and treatment were observed between the two groups of patients.

### 3.2. Clinical Impacts of Gene Mutation

The Oncomine Comprehensive Assay v1, a targeted NGS assay, was applied to the FFPE tumor samples to detect the presence of mutations across 143 genes. Associations between the individual mutated gene and the clinical outcome, with FDR-adjusted *p*-value < 0.1, are shown in Table 1 and Table 2. The mutation status of eight genes (*MET*, *U2AF1*, *BCL9*, *PPP2R1A*, *IDH2*, *CBL*, *BTK*, and *CHEK2*) were positively correlated with poor PFS and OS. In contrast, *IFITM1* and *DNMT3A* mutations were associated with better clinical outcomes. *MET* was selected for further studies because the adjusted *p*-value was the smallest, and the frequency of mutation was not low.

To eliminate the potential confounding effects, patients were stratified into groups of patients with and without *MET* mutation, and potential factors, including age, staging, histology, grading, and treatments, were compared between these two groups. As shown in Table S3, no significant difference of these factors was observed in these two groups. 

### 3.3. MET Mutation Is a Cancer Driver

**Figure 1:**
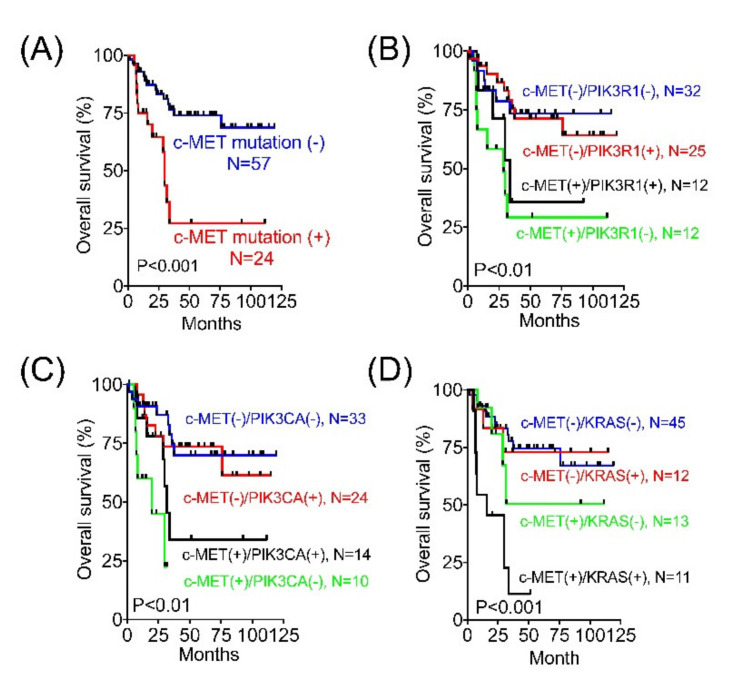
Impact of *MET* mutation and concurrent genetic mutations on the clinical outcome of advanced endometrial cancer. (**A**) Kaplan–Meier curves for overall survival (OS) in patients with and without *MET* mutation compared using the log-rank test. (**B**–**D**) Kaplan–Meier curves for OS were analyzed between the *MET* wild-type and *MET* mutant patients with or without concurrent *PIK3R1* (**B**), *PIK3CA* mutation (**C**), and *KRAS* (**D**).

*MET* mutation is a poor clinical marker (Figure 1A). c-MET, a protein encoded by the human *MET* gene, is a receptor tyrosine kinase (RTK) expressed on the cell surface [17]. The aberrant activation of the c-MET pathway and crosstalk with other RTKs has been shown to stimulate the PI3K/AKT and RAS/MAPK signaling pathways, which contribute to cancer biology (Figure S1A). *MET* mutation was associated with poor survival independent of EGFR mutation (Figure S1B). Alternatively, EGFR mutation did not affect the clinical outcome in patients harboring the wild-type or mutant *MET*. Similar results were observed in the *MET* and *ERBB2*, *MET* and *PIK3R1*, and *MET* and *PIK3CA* combination mutations (Figure S1C and Figure 1B,C). However, the impacts of *KDR* and *KRAS* mutations were different. The presence of *KDR* or *KRAS* mutations indicated poor outcomes in patients with *MET* mutations but not in those without *MET* mutations (Figure S1D and Figure 1D). Taken together, *MET* mutations highly influence the clinical outcome of advanced endometrial cancer, and *KDR* and *KRAS* mutations exhibit additional impacts on patients with a *MET* mutation.

### 3.4. In Silico Analysis of MET Mutations

**Figure 2:**
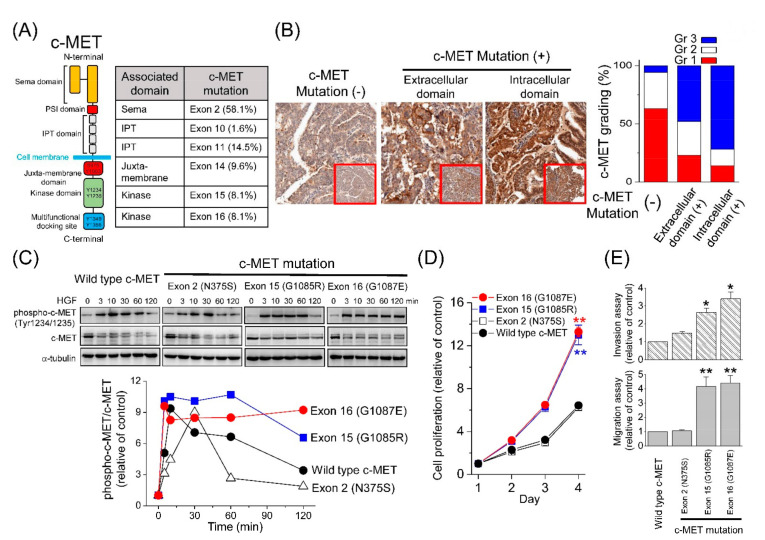
Differential effects of various *MET* mutations on c-MET phosphorylation and cellular functions. (**A**) The structure of c-MET (left) and distribution of *MET* mutations (right). Sema, semaphoring domain; PSI, plexin–semaphorin–integrin domain; IPT, immunoglobulin–plexin–transcription domain. (**B**) Representative images and grading of c-MET expression in endometrial cancer. The expression of c-MET was scored as grade 1–3, according to the percentage of the positively-stained tumor cells and the intensity of the staining. (**C**) Time course of c-MET phosphorylation in response to HGF (50 ng/mL) stimulation. Cell lysates from various clones of the endometrial cancer RL95-2 cells were immunoblotted with anti-c-MET, anti-phospho-c-MET, and anti-α-tubulin antibodies (upper). The expression levels were quantified, and the ratios of phosphor-c-MET to total c-MET are shown (bottom). (**D**) Cell proliferation, invasion, and migration. (**E**) Assays using various clones of the RL95-2 cells. Each value represents the mean ± standard error of mean (SEM) from at least three experiments. *, *p* < 0.05; **, *p* < 0.01.

The types of *MET* mutations were analyzed in 112 samples, including 81 primary tumors and 31 metastatic tissues. A total of 35 non-synonymous mutations, including missense mutations and small INDELs, were identified in exons 2, 10, 11, 14, 15, and 16 (Figure 2A and Figure S2A). Aberrant *MET* mutations resulted in c-MET overexpression in endometrial cancer tissues (Figure 2B), similar to that seen in lung cancer, indicating that c-MET overexpression alone can induce oncogenic transformation in vitro and in vivo [17,18,19,20]. Moreover, patients with intracellular domain mutations seemed to exhibit a worse outcome (Figure S2B). 

To predict the impact of the *MET* mutations on the protein structure, an in silico analysis was performed [21]. When the interaction between the semaphorin domain of c-MET and HGF was analyzed (Figure S3A), the exon 2 mutations did not cause any obvious change in the predicted free energy, implying that these mutations did not theoretically affect the binding of HGF to c-MET (Figure S3B). For mutations in the kinase domain, the free energy changes were studied in the presence of ATP (Figure S3C). As shown in Figure S3D, the in silico models demonstrated a Gly-to-Arg or Gly-to-Glu change at codon 1085 or a Gly-to-Glu change at codon 1087, which increased the mutational free energy. These findings indicate that these mutations might affect ATP binding, c-MET phosphorylation, and subsequent biological functions.

### 3.5. Effect of MET Mutations on Cellular Function

To confirm the in silico analysis, we established various clones of *MET* mutants in the RL95-2 and KLE cell lines. The time course of c-MET phosphorylation was studied in response to HGF stimulation. In the wild-type c-MET RL95-2 cells, HGF induced a rapid increase in c-MET phosphorylation within 3 min, which was gradually decreased over 120 min (Figure 2C). In contrast, an equivalent increase in c-MET phosphorylation was observed, which was sustained for 120 min in exons 15 (G1085E) or 16 (G1087E) in the clones of the kinase domain mutants. In the mutated semaphorin domain N375S clones, a delayed peak in c-MET phosphorylation was noted at 30 min, followed by rapid dephosphorylation. These results imply that different *MET* aberrations might exert differential effects on cellular functions [22,23]. This hypothesis was confirmed by proliferation, migration, and invasion assays in the endometrial cancer cell lines (Figure 2D,E and Figure S4). 

### 3.6. Endometrial Cancer Growth In Vivo

**Figure 3:**
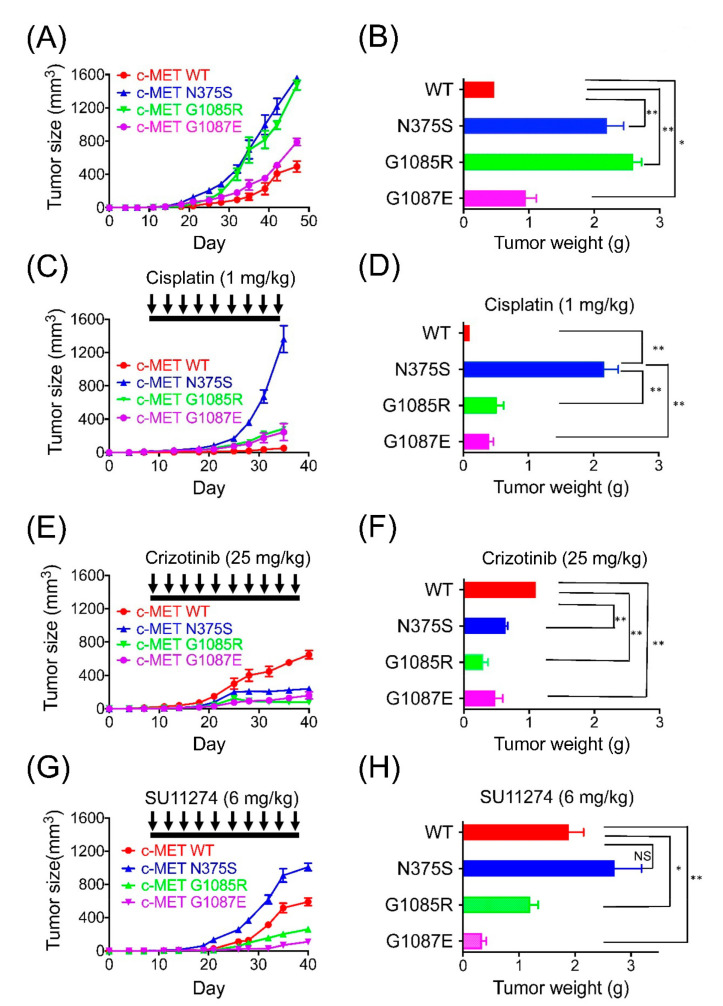
Antitumor effects of cisplatin and tyrosine kinase inhibitors in endometrial cancer xenografts harboring different *MET* mutations. 1 × 10^6^ RL95-2 cells carrying wild-type or mutant *MET*, including N375S, G1085R, and G1087E, were injected into the posterior flank of SCID mice subcutaneously. The tumors were allowed to grow and were treated by observation only (**A**,**B**), cisplatin (1 mg/kg) twice a week (**C**,**D**), crizotinib (25 mg/kg) by oral gavage twice a week (**E**,**F**), or SU11274 (6 mg/kg) twice a week (**G**,**H**) (*n* = 5 in each treatment group). Tumor size and body weight were measured twice a week. Data are presented as the mean tumor size ± SEM. *, *p* < 0.05; **, *p* < 0.01; NS, non-significance.

To test whether the c-MET signaling pathway was a therapeutic target, we subcutaneously inoculated the SCID mice with RL95-2 cells carrying various *MET* mutations. Tumors harboring different *MET* mutants showed a significant increase in volume when compared to those with wild-type *MET* (Figure 3A,B, and Figure S5). Although *MET* N375S did not have impacts on cell proliferation (Figure 2D), an increase in tumor size and weight was observed in tumors harboring the *MET* N375S mutation (Figure 3A,B). Next, cisplatin significantly inhibited the growth of tumors carrying the wild-type *MET* and the G1085R and G1087E mutations (Figure 3C,D). However, it appeared to exhibit no inhibitory effect on tumors with *MET* N375S. Crizotinib, a multi-target tyrosine kinase inhibitor, significantly inhibited the growth of tumors carrying the N375S, G1085R, or G1087E mutant compared to those with the wild-type *MET* (Figure 3E,F). SU11274, a specific c-MET inhibitor, also showed potent inhibitory effects on the growth of tumors carrying G1085R or 1087E mutations, whereas its inhibitory effect on the growth of tumors carrying wild-type *MET* was not very obvious. Interestingly, tumors with N375S mutation were insensitive to SU11274 in vivo (Figure 3G,H). These data indicate that *MET* mutations promote the growth of endometrial tumors and show different sensitivities to cisplatin or tyrosine kinase inhibitors (crizotinib and SU11274), depending on the type of *MET* mutation.

### 3.7. MET N375S Is a Germline Variant

**Figure 4:**
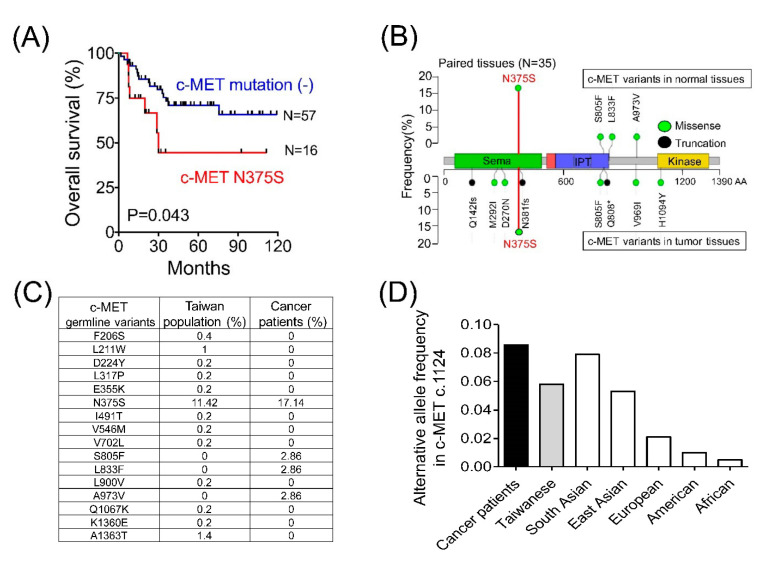
The clinical impact, distribution, and frequency of the germline *MET* N375S variant. (**A**) OS of advanced endometrial cancer patients with and without the *MET* N375S variant. (**B**) The distribution and frequency of *MET* variants in 35 paired normal and tumor tissues. Whole-genome sequencing was used to analyze DNA extracted from blood samples or adjacent non-tumor specimens to determine germline *MET* variants. (**C**) The distribution and frequency of germline *MET* variants in cancer patients and the normal Taiwan population. The database from the Taiwan Biobank, which contained germline whole-genome sequencing data of 499 normal Taiwanese individuals, was used to compare the distribution and frequency of germline *MET* variants in the NCKUH cohort and the normal Taiwan population. (**D**) The alternative allele frequency of *MET* c.1124 (N375S) in the different ethnic groups. The frequency of the variant allele in *MET* c.1124 was compared among the NCKUH cohort, the normal Taiwanese population, and different ethnic groups enrolled in the 1000 Genomes Project.

In animal studies, mutation of the semaphorin domain N375S displayed resistance to cisplatin, the major chemotherapeutic agent for endometrial cancer (Figure 3C,D). In our cohort, the OS rate for patients without *MET* mutations was 75%; however, that value significantly decreased to 50% in patients with *MET* N375S (Figure 4A, *p* = 0.043). Furthermore, an analysis of 35 paired tissues revealed that the *MET* N375S mutation was a germline variant (Figure 4B), consistent with the findings of a previous study in lung cancer [24]. When compared to other non-synonymous variants, the incidence of the N375S variant (c.1124 A > G) was relatively high (11~17%) in the current study and the Taiwan Biobank (Figure 4C). The worldwide distribution of genetic N375S variants (c.1124 A > G) was assessed in the data from the 1000 Genomes Project. The allele frequency of the *MET* N375S variant (c.1124 A > G) was 8.5% and 5.8% in all the patients enrolled in the present study and the Taiwan Biobank, respectively. A similar allele frequency (5~8%) was observed in the population from South and East Asia (Figure 4D). In contrast, less than 2% of the alternative allele frequency was observed in the European, American, and African populations.

### 3.8. Comparison with TCGA Molecular Classification

Patients with advanced disease (stage III or IV) in the TCGA endometrial cancer cohort were identified, and the genomic backgrounds between the NCKUH and TCGA cohorts were compared. No significant differences in age distribution at initial diagnosis, FIGO stage, histology, and clinical outcome were observed between the two cohorts (Figure S6A,B). To obtain somatic mutations, the variants identified by Oncomine Comprehensive Assay v1 were filtered by the germline genetic variants. The top gene aberrations, with high impact on genomic functions, were listed after analyzing and filtering out the mutations, which were categorized as “low” (harmless or unlikely to change protein behavior), “modifier” (affecting the non-coding), or “moderate” (inframe insertion or protein-altering variant; Figure S6C). The ranking of the top 10 genetic aberrations was different between the two cohorts, although *PTEN* was the most common mutated gene. Mutations in *BRAF*, *JAK1*, and *KIT* were identified in approximately 4–11% of the patients in the TCGA cohort, but they were rarely detected in the NCKUH cohort. In addition, at the advanced stage, *MET* mutations were observed in 15% of cases in the TCGA cohort without an impact on OS (Figure S6D).

A subset of endometrioid cancer was newly identified in hotspot *POLE* mutations in the TCGA cohort [5]. TCGA and subsequent studies showed that POLE-mutant endometrial cancers typically present as high-grade or poorly differentiated tumors [25]. In addition, *POLE* mutations accompanied by an ultra-tumor mutation burden present favorable clinical outcomes [26]. Among the 81 patients recruited in this study, 74 presented adequate and qualified samples to investigate the pathogenic mutations in the *POLE* gene. However, no association between the *POLE* mutation and the histology of the specimens was observed in the current study cohort (Figure S7A). In addition, OS was not affected by the presence of the *POLE* mutation (Figure S7B). A discrepancy in the genetic aberrations between the NCKUH and TCGA cohorts was noted.

## 4. Discussion

This study highlights the survival-associated molecular pathways in advanced endometrial cancer. *MET* mutation was found to be a potential therapeutic target. This conclusion was reached based on several important findings. First, the *MET* mutation was noted in 30% of advanced endometrial cancer cases and was associated with a poor clinical outcome; concurrent *MET* and *KRAS* mutations indicated the worst outcome. Second, *MET* mutation promotes the growth and invasion of endometrial tumors both in vivo and in vitro. Third, *MET* mutation occurs in the HGF-binding or kinase domain. Mutations in the kinase domain induce sustained c-MET phosphorylation in response to HGF stimulation. Consistently, patients with kinase domain mutations presented with the worst clinical outcomes compared to those with mutations in extracellular regions. Fourth, tumors harboring c-MET kinase domain mutations in the SCID mouse model were sensitive to the tyrosine kinase inhibitors and cisplatin. Finally, mutation of Sema domain N375S provided resistance against cisplatin, and this effect was not overcome by the selective c-MET inhibitor. 

The HGF-MET signaling pathway regulates cell proliferation and motility. HGF is a complex consisting of six domains, including an N-terminal domain, four kringle domains, and a C-terminal serine proteinase homology domain. After the site-specific proteolysis, alpha and beta chains were produced to form the mature HGF heterodimer. The Sema domain of c-MET is necessary for HGF-binding, receptor dimerization, and the activation of the downstream signaling. In the present study, 58% of *MET* mutations were identified in the Sema domain, and N375S was the most frequent mutation within this region. An analysis of paired genomic DNA in normal and tumor tissues and in the data obtained from the 1000 Genomes Project revealed that the *MET* N375S mutation was a germline variant with high frequency in the Asian population. This ethnic difference was previously reported in patients with lung cancer, and *MET* N375S was reported to confer resistance to c-MET inhibition [24]. Earlier studies suggested that resistance to the c-MET inhibitor was caused by a missense change in the 375 serine residue, leading to a weakening in the interaction between HGF and c-MET and a decrease in kinase activation, thereby resulting in increased resistance to the c-MET inhibitor [16,27]. This notion was supported by our experiments in the endometrial cancer cell line, which showed that HGF induced delayed c-MET phosphorylation, accompanied by rapid dephosphorylation. In the SCID mouse models, tumors harboring *MET* N375S were consistently resistant to the specific c-MET inhibitor, SU11274. 

Interestingly, *MET* N375S provided drug resistance to cisplatin in the mouse model in the current study. Cisplatin is one of the most active chemotherapeutic agents used in endometrial cancer. The poor clinical outcome in patients with the *MET* N375S mutation in the NCKUH cohort supported this important finding in vivo. To the best of our knowledge, this correlation with chemoresistance has not been reported so far. Given the nature of the germline genetic variant, N375S would be a de novo mechanism of cisplatin resistance. The selection of patients based on the germline *MET* genetic variant may prove more beneficial for patients with endometrial cancer receiving cisplatin-based therapy and could be a new therapeutic strategy in precision medicine.

In this study, co-occurrence of the *MET* and *PIK3CA* or *KRAS* mutations was detected in 14 and 11 of 81 patients, respectively. The *MET*/*PIK3CA* and *MET*/*KRAS* co-mutation may have biological impacts on endometrial cancer cells. First, mutations in *PIK3CA* and *KRAS* cause the aberrant activation of downstream signaling pathways, contributing to tumorigenesis and disease progression in endometrial cancer [28,29]. The co-occurrence of *MET* and *KRAS* or *MET* and *PIK3CA* might increase the aggressiveness of cancer cells, including cell proliferation, survival, invasion, or metastasis. This hypothesis was supported by clinical data showing that patients with the *MET* and *KRAS* co-mutation had the worst outcome (Figure 1D). Further in vitro and in vivo studies are still needed to confirm this hypothesis. Second, our data showed *MET* is a potential therapeutic target for endometrial cancer. Currently, a clinical trial testing the efficacy of c-MET inhibitors in patients with advanced endometrial cancer is on-going (https://clinicaltrials.gov/ct2/show/NCT04030429, accessed on 24 July 2019). The *KRAS* mutation has been shown to be one of the resistant mechanisms in *MET*-mutant non-small cell lung cancer patients receiving the c-MET tyrosine kinase inhibitor [30]. The co-occurrence of *MET* and *PIK3CA* or *KRAS* mutations might also mediate the resistance to c-MET inhibitors when targeting the *MET* mutation in endometrial cancer. 

A discrepancy in the genetic aberration in terms of the severity of the effect of the mutation on genomic functions was observed between the current study cohort and the TCGA cohort. In the TCGA cohort, *POLE*-mutant endometrial cancers were typically high-grade or poorly differentiated [5]. *POLE* mutations accompanied by an ultra-tumor mutation burden presented with a favorable clinical outcome [26]. Conversely, in our cohort, the results showed no associations between the *POLE* mutation, histologic subtype, and clinical outcome. 

The mutational analysis in this study was performed by a deep targeted gene sequencing assay, the Oncomine Comprehensive Assay version 1, instead of whole exome (WES) or whole-genome sequencing (WGS). The analyses of the *POLE* gene and microsatellite instability were not included in this assay, and the data of copy number alterations were only available for 26 genes. Although the mutations in the *POLE* gene could be analyzed in 74 patients who had adequate tumor samples, stratification of these 81 endometrial cancer patients into 4 groups by *POLE* mutation, MSI status, and copy number cluster based on TCGA’s classification still could not be achieved. Therefore, the impact of *MET* mutations in the four subgroups remains unclear. 

## 5. Conclusions

This integrated genomic and mechanistic study provides insights into the biological and diagnostic classification of advanced endometrial cancer, which might result in a direct effect on treatment recommendations for patients with this disease. Furthermore, this information provides opportunities for additional genome-guided clinical trials and drug development. Since several c-MET inhibitors are FDA-approved drugs and in the late phases of the clinical trials, the next step is to launch a clinical trial targeting *MET* mutations in advanced endometrial cancers.

## Figures and Tables

**Table 1:** The association between the mutated genes and overall survival.

Gene	Case Number with Mutation	Case Number without Mutation	*p*-Value	Adjusted *p*-Value	Hazard Ratio	95% Interval (Lower~Upper)
*MET*	24	57	0.019	0.067	2.606	1.167–5.819
*U2AF1*	3	78	0.006	0.067	5.942	1.683~20.980
*BCL9*	4	77	0.022	0.067	4.343	1.241~15.201
*PPP2R1A*	8	73	0.020	0.067	3.272	1.204~8.892
*IFITM1*	79	2	0.018	0.067	0.151	0.031~0.724
*IDH2*	2	79	0.018	0.067	6.44	1.381~30.036
*EGFR*	7	74	0.041	0.088	2.785	1.040~7.457
*CBL*	2	79	0.039	0.088	4.856	1.086~21.703
*DNMT3A*	9	72	0.049	0.088	0.222	0.049~0.995
*BTK*	2	79	0.039	0.088	4.856	1.086~21.703
*RB1*	21	60	0.079	0.094	2.012	0.923~4.382
*CHEK2*	4	77	0.067	0.094	3.215	0.921~11.230
*MLH1*	14	67	0.077	0.094	2.216	0.918~5.348
*PIK3R1*	37	44	0.065	0.094	2.113	0.954~4.680
*ESR1*	2	79	0.058	0.094	7.715	0.937~63.546
*KRAS*	37	44	0.068	0.094	2.059	0.949~4.469
*FGFR3*	7	74	0.083	0.094	2.621	0.882~7.792
*GNAQ*	3	78	0.089	0.097	3.749	0.818~17.190
*NKX2_1*	3	78	0.098	0.098	3.615	0.790~16.551
*CCNE1*	5	76	0.097	0.098	2.572	0.843~0.098

**Table 2:** The association between the mutated genes and progression-free survival.

Gene	Case Number with Mutation	Case Number without Mutation	*p*-Value	Adjusted *p*-Value	Hazard Ratio	95% Interval (Lower~Upper)
*MET*	24	57	0.044	0.067	2.081	1.020~4.248
*BCL9*	4	77	0.059	0.067	3.274	0.958~11.194
*TSC2*	27	54	0.029	0.067	2.122	1.079~4.177
*NF1*	48	33	0.052	0.067	2.222	0.993~4.974
*MAP2K2*	2	79	0.063	0.067	4.066	0.926~17.854
*NFE2L2*	6	75	0.062	0.067	0.244	0.056~1.073
*U2AF1*	3	78	0.008	0.067	5.395	1.563~18.615
*CHEK2*	4	77	0.049	0.067	3.503	1.007~12.184
*VHL*	8	73	0.020	0.067	3.293	1.206~8.993
*CBL*	2	79	0.016	0.067	6.589	1.425~30.475
*IFITM1*	79	2	0.023	0.067	0.168	0.036~0.778
*IDH2*	2	79	0.049	0.067	4.398	1.005~19.250
*DNMT3A*	9	72	0.036	0.067	0.267	0.078~0.918
*BTK*	2	79	0.016	0.067	6.589	1.425~30.475
*PPP2R1A*	8	73	0.087	0.087	2.329	0.883~6.143

## Data Availability

The datasets generated and analyzed during the current study will be uploaded to NCBI.

## Supplementary Materials

The following are available online at https://www.mdpi.com/article/10.3390/cancers13164231/s1. Supplementary Figure S1. Impact of *MET* mutation and concurrent genetic mutations on clinical outcome of advanced endometrial cancer. (A) Crosstalk between c-MET and other membrane receptors activated important downstream signaling. (B–D) Kaplan–Meier curves for overall survival were analyzed among *MET* wild-type and *MET* mutant patients with or without concurrent *EGFR* (B), *ERBB2* (C), or *KDR* (D). Supplementary Figure S2. *MET* mutational patterns and overall survival. (A) All variants of *MET* mutations were analyzed from 112 surgical samples of advanced endometrial cancer, including 81 primary tumors (P) and 31 metastatic tissues (M). (B) The relationship between *MET* mutational patterns and overall survival. Supplementary Figure S3. The potential effects of *MET* mutations on protein structure. (A,B) In silico analysis to study the interaction between the semaphorin domain of c-MET and HGF. (C,D) The free energy changes were studied in the presence of ATP for the 10 *MET* mutations located in the kinase domain. Supplementary Figure S4. Effect of *MET* mutations on the cellular function of the endometrial cancer KLE cell line. (A,B) A representative image and quantitative analysis of invasion and migration assays on various clones of *MET* mutation. (C) Proliferation assay. Each value represents mean ± SEM from at least 3 different samples. * *p* < 0.05; ** *p* < 0.01. Supplementary Figure S5. SCID mice were subcutaneously inoculated with endometrial cancer RL95-2 cells carrying either wild-type or mutant *MET* (N375S, G1085R, or G1087E). The mice were treated with observation alone (A), cisplatin 1 mg/kg (B), crizotinib 25 mg/kg (C), or SU1127 6 mg/kg (D) 1 week after tumor inoculation. The tumor size at the day when mice were sacrificed is shown. Supplemental Figure S6. Comparison between TCGA and NCKUH cohorts. (A) Distribution of ages at initial diagnosis, FIGO stages, histologic subtypes in TCGA and NCKUH cohorts. (B) The clinical outcome of TCGA and NCKUH cohorts. There was no significant difference in these parameters between the two cohorts. (C) High impact of gene aberration on genomic function. Top 23 genes with high impact of genomic alterations are listed in each cohort. (D) The overall survival rate was not affected by *MET* mutations in the TCGA cohort. Supplementary Figure S7. DNA polymerase epsilon (POLE) mutations in advanced endometrial cancer. (A) Mutation patterns of POLE in the NCKUH cohort. (B) Overall survival in the presence or absence of the POLE mutation in the NCKUH cohort. Supplementary Figure S8. The uncropped Western blot figures of Figure 2C. Supplementary Table S1. Sequencing coverage and quality statistics of next-generation sequencing. Supplementary Table S2. Patient characteristics. Supplementary Table S3. The clinical characteristics of patients with and without *MET* mutation.
