# Supplementary material for: MET Mutation Is a Potential Therapeutic Target for Advanced Endometrial Cancer
Source: Cancers (Basel). 2021 Aug 23;13(16):4231. doi: 10.3390/cancers13164231 (PMC8392057; doi:10.3390/cancers13164231)
Supplement: Supplementary file 1 [file cancers-13-04231-s001.zip › cancers-1323092-supplementary-proof/supplementary/Supplementary Figure S1.pdf]

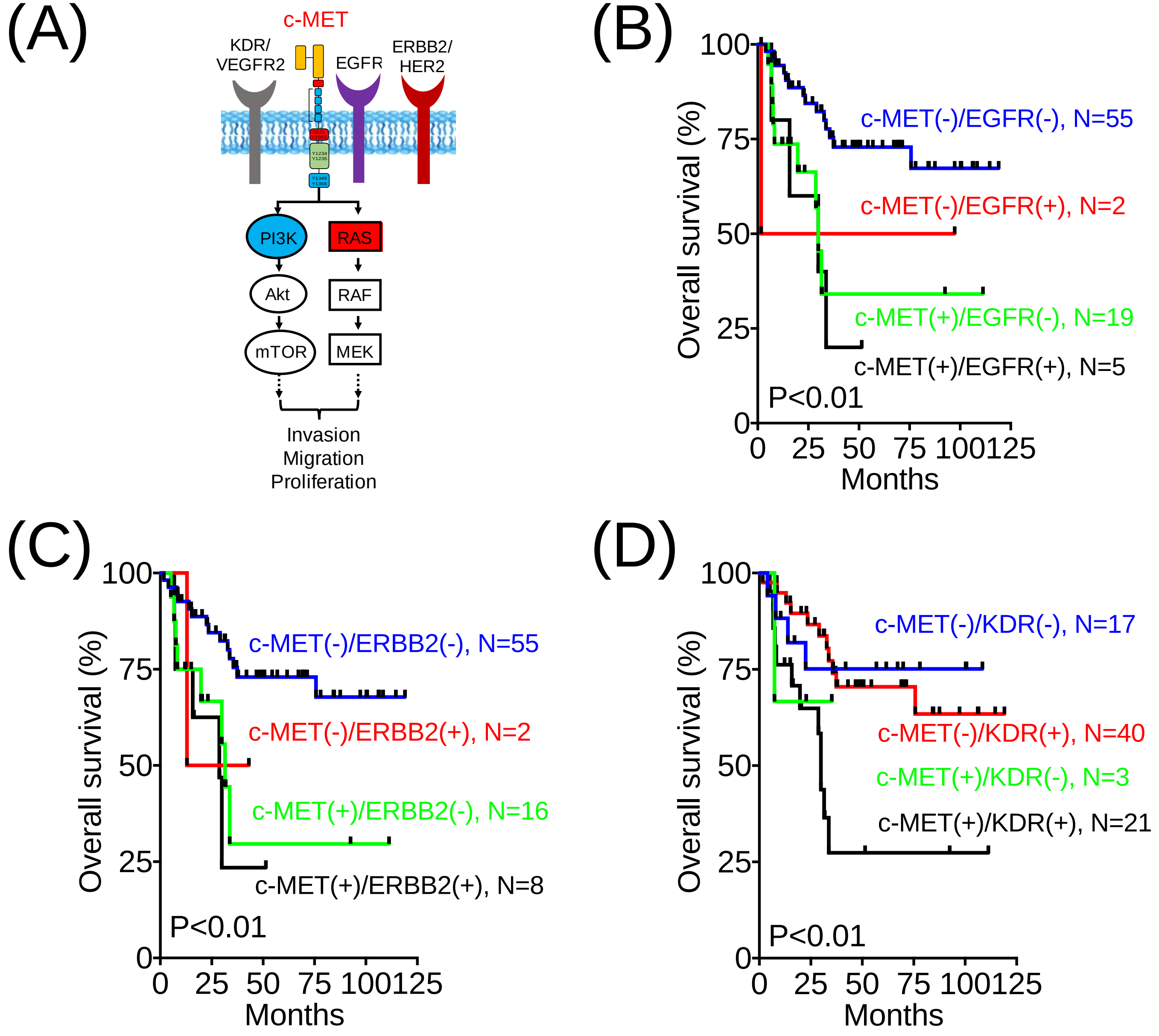

Supplementary Figure S1. Impact of *MET* mutation and concurrent genetic mutations on clinical outcome of advanced endometrial cancer. (A) Crosstalk between c-MET and other membrane receptors activated important downstream signaling. **(B~D)** Kaplan-Meier curves for overall survival were analyzed among *MET* wild type and *MET* mutant patients with or without concurrent *EGFR* **(B)**, *ERBB2* **(C)**, or *KDR* **(D)**.
