# Supplementary material for: MET Mutation Is a Potential Therapeutic Target for Advanced Endometrial Cancer
Source: Cancers (Basel). 2021 Aug 23;13(16):4231. doi: 10.3390/cancers13164231 (PMC8392057; doi:10.3390/cancers13164231)
Supplement: Supplementary file 1 [file cancers-13-04231-s001.zip › cancers-1323092-supplementary-proof/supplementary/Supplementary Figure S2.pdf]

(A)

	c-MET exon	Protein domain	c-MET mutation		Tumor tissue
Extracellular domain	Exon 2 (58.1%)	Sema domain	A82V	(N = 2)	P/M
			E91K	(N = 1)	P
			P97S	(N = 1)	M
			D100N	(N = 1)	M
			Q142fs	(N = 1)	P
			D270N	(N = 1)	M
			M292I	(N = 1)	M
			P351L	(N = 1)	M
			N375S	(N = 24)	P/M
			N381fs	(N = 2)	M
			Y390fs	(N = 1)	M
	Exon 10 (1.6%)	IPT domain	R784K	(N = 1)	M
	Exon 11 (14.5%)	IPT domain	C800Y	(N = 1)	P
			S805F	(N = 2)	P/M
			Q808*	(N = 2)	P
			A819V	(N = 1)	P
			G825R	(N = 1)	P
			L827F	(N = 1)	M
			L833F	(N = 1)	P
Intracellular domain	Exon 14 (9.6%)	Juxta-membrane domain	V969I	(N = 1)	P
			R970C	(N = 1)	M
			D972N	(N = 1)	P
			A973V	(N = 1)	M
			S988N	(N = 1)	P
			T993A	(N = 1)	P
	Exon 15 (8.1%)	Kinase domain	H1079Y	(N = 1)	P
			E1082K	(N = 1)	M
			G1085R	(N = 1)	M
			G1085E	(N = 1)	M
			R1086K	(N = 1)	P
	Exon 16 (8.1%)	Kinase domain	G1087E	(N = 1)	M
			H1088Y	(N = 1)	P
			H1094Y	(N = 1)	M
			T1096I	(N = 1)	P
			C1107Y	(N = 1)	M

(B)

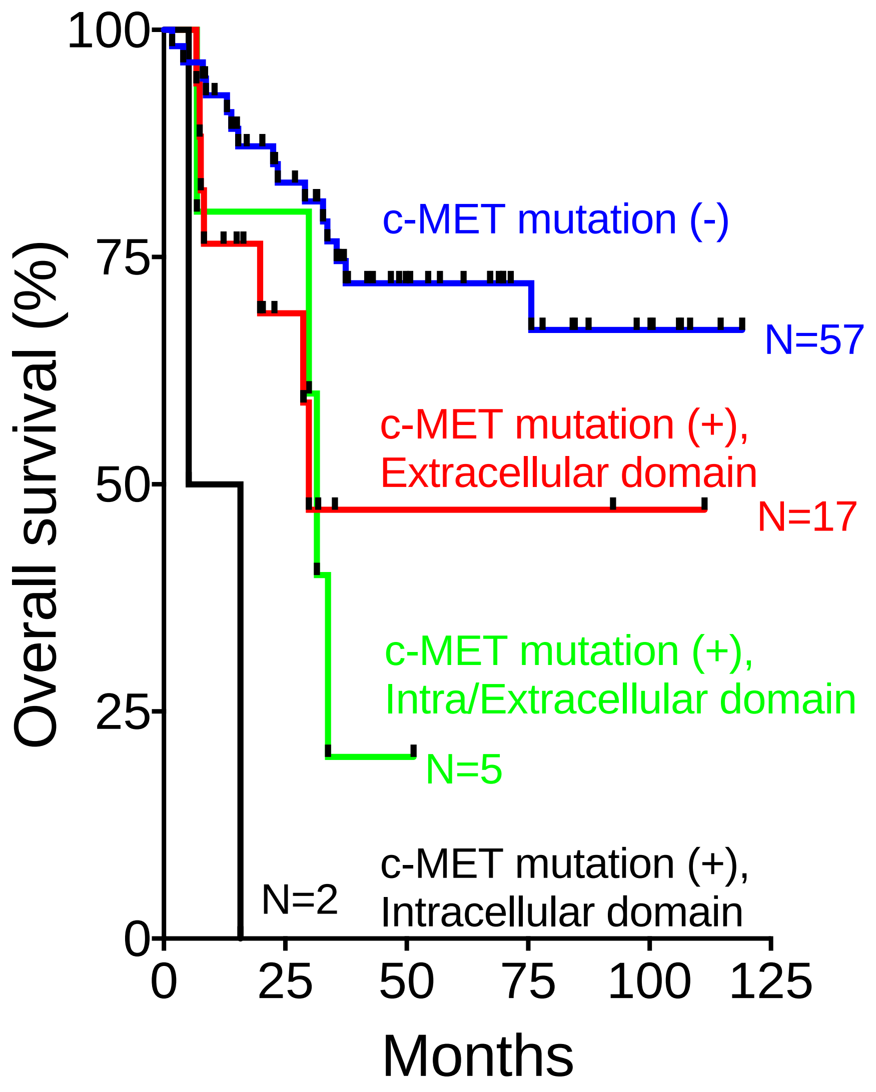

Supplementary Figure S2. *MET* mutational patterns and overall survival. (A) All variants of *MET* mutations were analyzed from 112 surgical samples of advanced endometrial cancer, including 81 primary tumors (P) and 31 metastatic tissues (M). **(B)** The relationship between *MET* mutational patterns and overall survival.
