# Supplementary material for: MET Mutation Is a Potential Therapeutic Target for Advanced Endometrial Cancer
Source: Cancers (Basel). 2021 Aug 23;13(16):4231. doi: 10.3390/cancers13164231 (PMC8392057; doi:10.3390/cancers13164231)
Supplement: Supplementary file 1 [file cancers-13-04231-s001.zip › cancers-1323092-supplementary-proof/supplementary/Supplementary Figure S3.pdf]

(A)

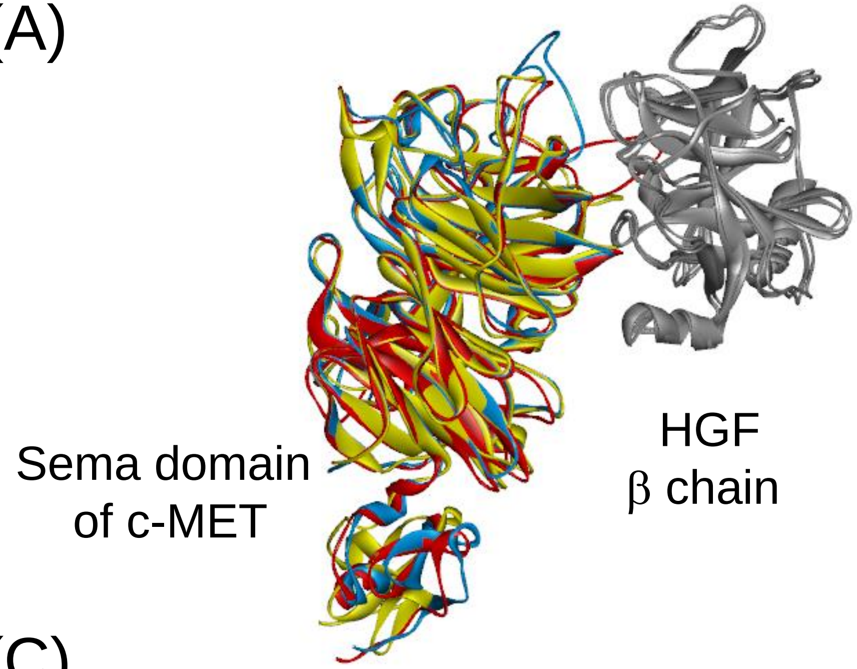

(B)

Sema Mutation	Mutation energy	Effect of mutation	VDW term	Electrostatic term	Entropy term	Nonpolar term
A82V	0	NEUTRAL	0	0	0	0
E91K	0.44	NEUTRAL	0	0.88	0	0
P97S	0.01	NEUTRAL	0	0.01	0	0
D100N	0.23	NEUTRAL	0	0.46	0	0
D270N	0.16	NEUTRAL	0	0.32	0	0
M292I	0	NEUTRAL	0	0	0	0
P351S	0.13	NEUTRAL	0	0.26	0	0
N375S	0	NEUTRAL	0	0	0	0

(C)

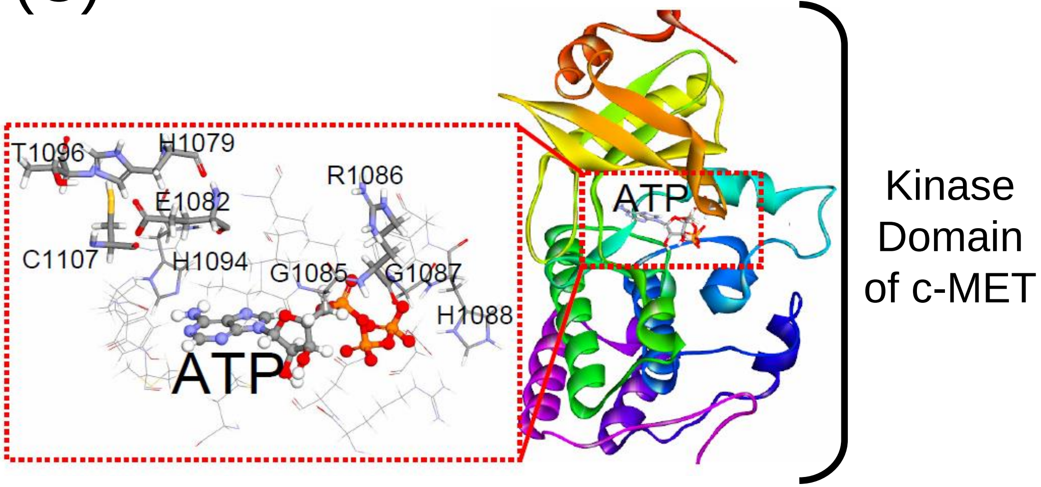

(D)

Mutation	Mutation energy	Effect of mutation	VDW term	Electrostatic term	Entropy term	Nonpolar term
H1079Y	0.03	NEUTRAL	0.05	0	0	0
E1082K	-0.01	NEUTRAL	0.01	-0.03	0	0
G1085R	3.02	DESTABILIZING	4.27	0.11	1.04	0
G1085E	0.63	DESTABILIZING	-0.15	0.5	0.57	0
R1086K	-0.12	NEUTRAL	-0.22	-0.02	0	0
G1087E	1.62	DESTABILIZING	3.22	-0.1	0.07	0
H1088Y	-0.25	NEUTRAL	-0.12	-0.36	-0.01	0
H1094Y	-0.08	NEUTRAL	-0.11	0	-0.03	0
T1096I	-0.01	NEUTRAL	-0.01	0	0	0
C1107Y	-0.1	NEUTRAL	-0.21	0	0	0

Supplementary Figure S3. The potential effects of *MET* mutations on protein structure. (A) & (B) *In silico* analysis was to study the interaction between the semaphorin domain of c-MET and HGF. **(C) & (D)** The free energy changes were studied in the presence of ATP for the 10 *MET* mutations located in the kinase domain.
