# Supplementary material for: MET Mutation Is a Potential Therapeutic Target for Advanced Endometrial Cancer
Source: Cancers (Basel). 2021 Aug 23;13(16):4231. doi: 10.3390/cancers13164231 (PMC8392057; doi:10.3390/cancers13164231)
Supplement: Supplementary file 1 [file cancers-13-04231-s001.zip › cancers-1323092-supplementary-proof/supplementary/Supplementary Figure S4.pdf]

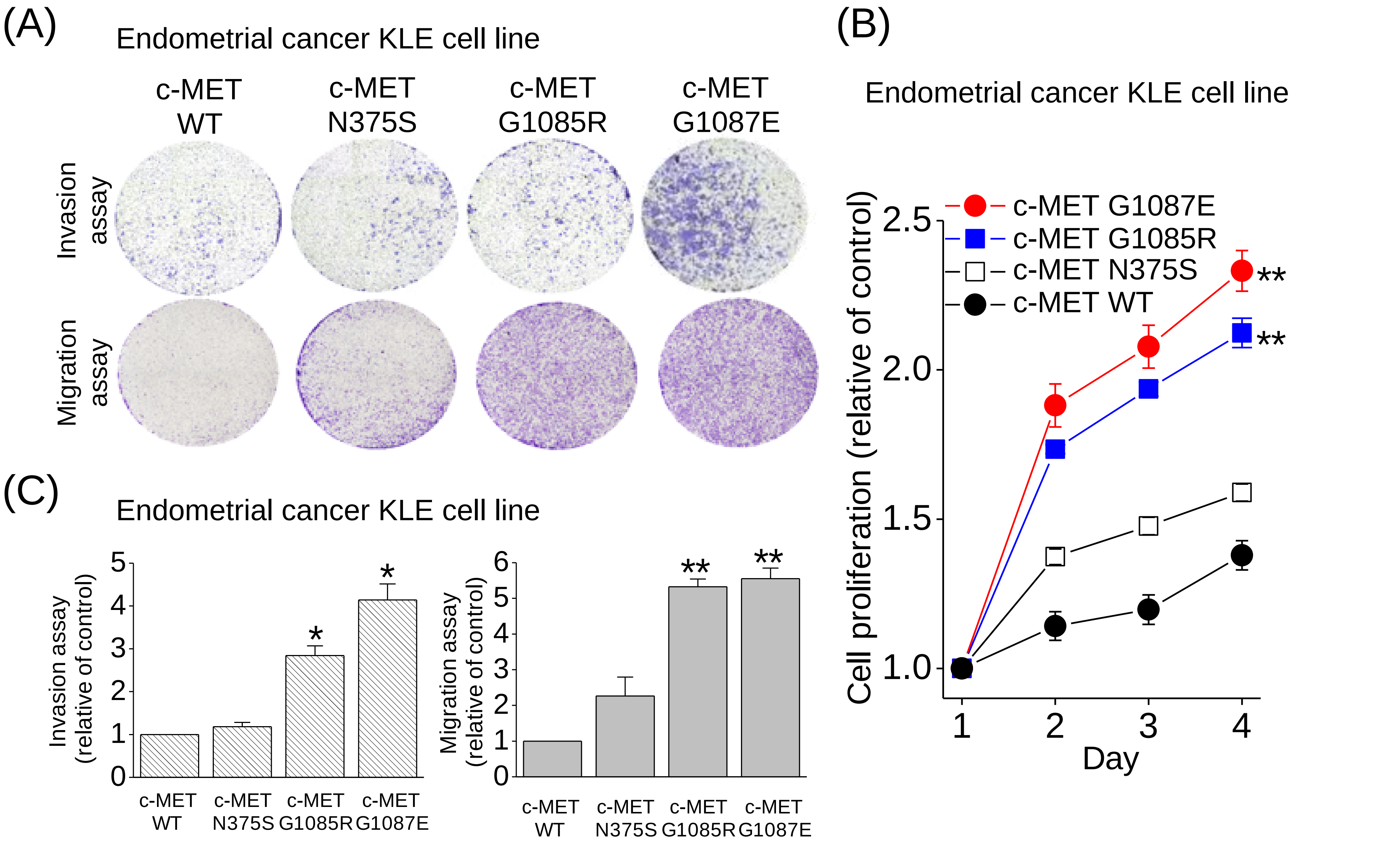

Supplementary Figure S4. Effect of *MET* mutations on the cellular function of endometrial cancer KLE cell line. (A) & (B) A representative image and quantitative analysis of invasion and migration assay on various clones of *MET* mutation. **(C)** Proliferation assay. Each value represents mean ± SEM from at least 3 different samples. *P < 0.05; **P < 0.01.
