# Supplementary material for: MET Mutation Is a Potential Therapeutic Target for Advanced Endometrial Cancer
Source: Cancers (Basel). 2021 Aug 23;13(16):4231. doi: 10.3390/cancers13164231 (PMC8392057; doi:10.3390/cancers13164231)
Supplement: Supplementary file 1 [file cancers-13-04231-s001.zip › cancers-1323092-supplementary-proof/supplementary/Supplementary Figure S5.pdf]

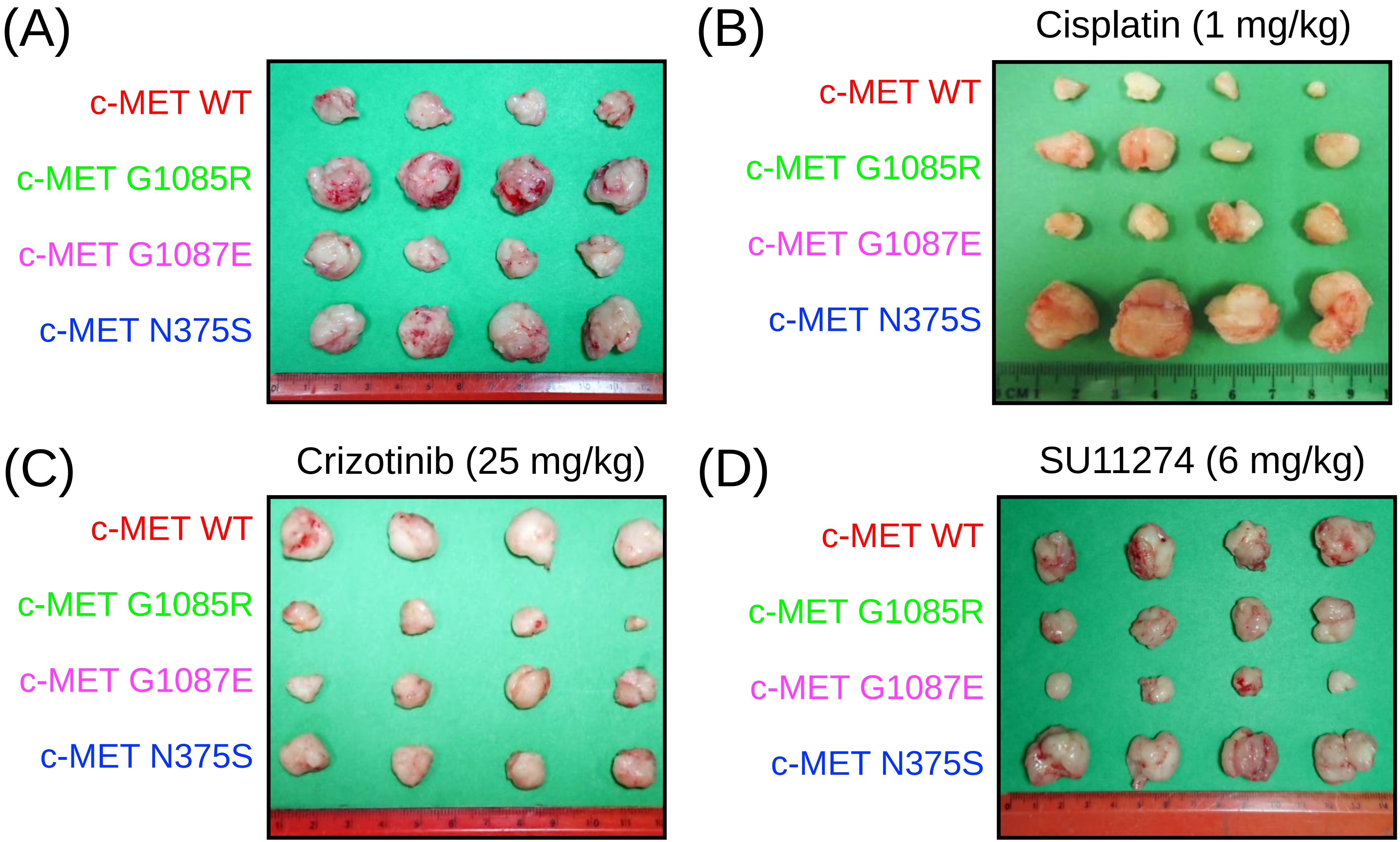

Supplementary Figure S5. SCID mice were subcutaneously inoculated with endometrial cancer RL95-2 cells carrying either wild type or mutant *MET* (N375S, G1085R, or G1087E). The mice were treated with observation alone **(A)**, cisplatin 1 mg/kg **(B)**, crizotinib 25 mg/kg **(C)**, or SU1127 6 mg/kg **(D)** 1 week after tumor inoculation. The tumor size at the day when mice were sacrificed was shown.
