# Supplementary material for: MET Mutation Is a Potential Therapeutic Target for Advanced Endometrial Cancer
Source: Cancers (Basel). 2021 Aug 23;13(16):4231. doi: 10.3390/cancers13164231 (PMC8392057; doi:10.3390/cancers13164231)
Supplement: Supplementary file 1 [file cancers-13-04231-s001.zip › cancers-1323092-supplementary-proof/supplementary/Supplementary Figure S6.pdf]

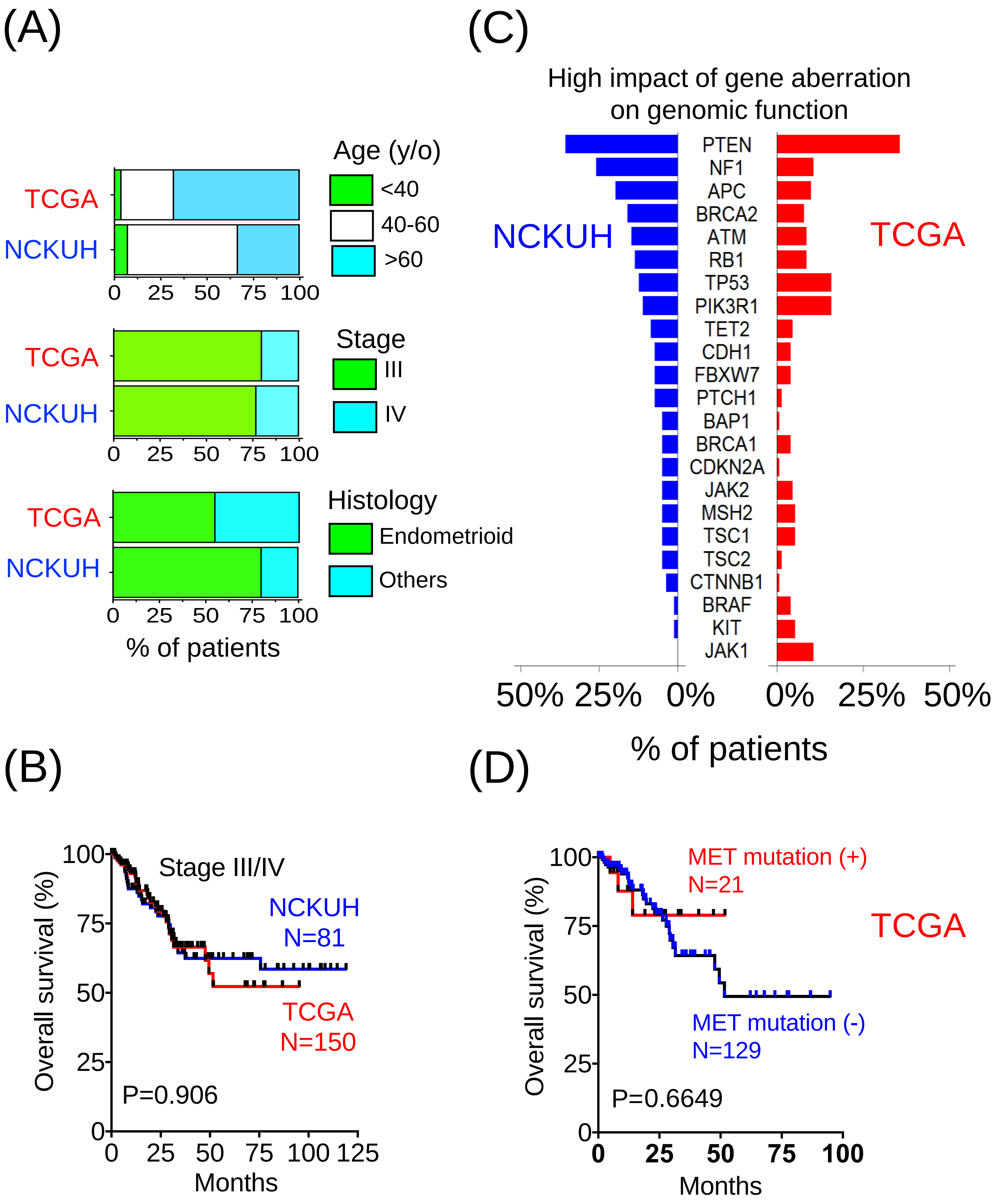

Supplemental Figure S6. Comparison between TCGA and NCKUH cohort. (A) Distribution of ages at initial diagnosis, FIGO stages, histologic subtypes in TCGA and NCKUH cohort. **(B)** The clinical outcome of TCGA and NCKUH cohort. There was no significant difference in these parameters between two cohorts. **(C)** High impact of gene aberration on genomic function. Top 23 genes with high impact of genomic alterations were listed in each cohort. **(D)** The overall survival rate was not affected by *MET* mutations in TCGA cohort.
