# Supplementary material for: MET Mutation Is a Potential Therapeutic Target for Advanced Endometrial Cancer
Source: Cancers (Basel). 2021 Aug 23;13(16):4231. doi: 10.3390/cancers13164231 (PMC8392057; doi:10.3390/cancers13164231)
Supplement: Supplementary file 1 [file cancers-13-04231-s001.zip › cancers-1323092-supplementary-proof/supplementary/Supplementary Figure S7.pdf]

(A)

Case	Exon 9 c.C857G	Exon9 c.C890T	Exon13 c.G1231T	Exon13 c.G1366C	Exon14 c.C1376T	Exon14 c.C1394T	Stage	Grade	Histology
	POLE mutation								
1		X				X	4	3	Endometroid
2		X				X	4	2	Endometroid
3		X					3	3	Endometroid
4				X			3	3	Endometroid
5	X						3	3	Endometroid
6	X						3	2	Endometroid
7			X		X		4	3	Endometroid
8					X		3	1	Endometroid
9					X		4	3	Endometroid

(B)

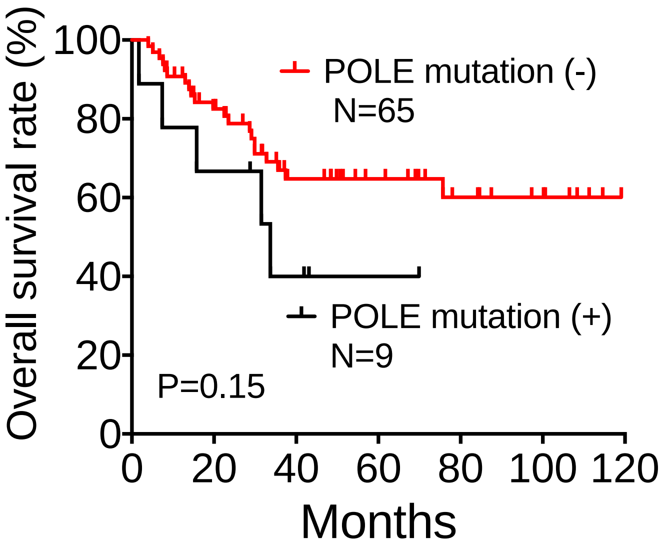

Supplementary Figure S7. DNA polymerase epsilon (POLE) mutations in advanced endometrial cancer. (A) Mutation patterns of POLE in NCKUH cohort. **(B)** Overall survival in the presence or absence of POLE mutation in NCKUH cohort.
