# Supplementary material for: MET Mutation Is a Potential Therapeutic Target for Advanced Endometrial Cancer
Source: Cancers (Basel). 2021 Aug 23;13(16):4231. doi: 10.3390/cancers13164231 (PMC8392057; doi:10.3390/cancers13164231)
Supplement: Supplementary file 1 [file cancers-13-04231-s001.zip › cancers-1323092-supplementary-proof/supplementary/Supplementary Table S2 20210804 by Yeh.pdf]

Table S2. Patient characteristics.

Characteristics	No evidence of disease (N=47)	Progressive disease or death (N=34)	*P*
Age (years)			
Mean (range)	53.2 (34 ~ 76)	58.4 (34 ~ 87)	0.025
FIGO staging			< 0.001
Stage III	44 (93.6 %)	18 (52.9 %)	
Stage IV	3 (6.4 %)	16 (47.1 %)	
Histology			0.115
Endometrioid	41 (87.2 %)	24 (70.6 %)	
Others	6 (12.8 %)	10 (29.4 %)	
Grading*			0.048
1	10 (21.3 %)	3 (8.8 %)	
2	23 (48.9 %)	12 (35.3 %)	
3	14 (29.8 %)	19 (55.9 %)	
Survival (months)			
PFS	54.0 (1.7 ~ 119.1)	18.4 (1.7 ~ 75.7)	< 0.001
OS	54.0 (1.7 ~ 119.1)	27.3 (1.7 ~ 92.5)	
Treatment			0.139
Staging surgery	47 (100 %)	31 (91.2 %)	
Others#	0 (0 %)	3 (8.8 %)	
Chemotherapy**			> 0.999
No	1 (2.1 %)	0 (0 %)	
Yes	46 (97.9 %)	34 (100 %)	

*: clear cell carcinoma, serous carcinoma, carcinosarcoma and mixed type carcinoma were classified as grade 3; #: dilation & curettage for diagnosis alone; PFS: progression-free survival; OS: overall survival. **: chemotherapy: 8 cases (9.8%) received the regimen of paclitaxel plus platinum; 72 cases (88.9%) received the combination of paclitaxel, doxorubicin and platinum.

Commented [M1]: Please confirm the red color is necessary, if yes, please define
