# Supplementary material for: MET Mutation Is a Potential Therapeutic Target for Advanced Endometrial Cancer
Source: Cancers (Basel). 2021 Aug 23;13(16):4231. doi: 10.3390/cancers13164231 (PMC8392057; doi:10.3390/cancers13164231)
Supplement: Supplementary file 1 [file cancers-13-04231-s001.zip › cancers-1323092-supplementary-proof/supplementary/Supplementary Table S3 20210804 by Yeh.pdf]

Table S3. The clinical characteristics of patients with and without *MET* mutation.

	Patients with *MET* mutation (N=24)	Patients without *MET* mutation (N=57)	*P* value
Age (years)			
Mean (SD)	55.6 (10.8)	55.3 (10.4)	0.904
FIGO staging - no. (%)			0.283
Stage III	16 (66.7)	46 (80.7)	
Stage IV	8 (33.3)	11 (19.3)	
Histology			0.883
Endometrioid	20 (83.3 %)	45 (78.9 %)	
Others	4 (16.7 %)	12 (21.1 %)	
Grading			0.848
1	3 (12.5 %)	10 (17.5 %)	
2	11 (45.8 %)	24 (42.1 %)	
3	10 (41.7 %)	23 (40.4 %)	
Treatment - no. (%)			>0.999
Staging surgery	23 (95.8)	55 (96.5)	
Others	1 (4.2)	2 (3.5)	
Chemotherapy - no. (%)			>0.999
Yes	24 (100)	56 (98.2)	
No	0 (0)	1 (1.8)	

SD, standard deviation.
