# Supplementary figures and images for: MET Mutation Is a Potential Therapeutic Target for Advanced Endometrial Cancer
Source: Cancers (Basel). 2021 Aug 23;13(16):4231. doi: 10.3390/cancers13164231 (PMC8392057; doi:10.3390/cancers13164231)

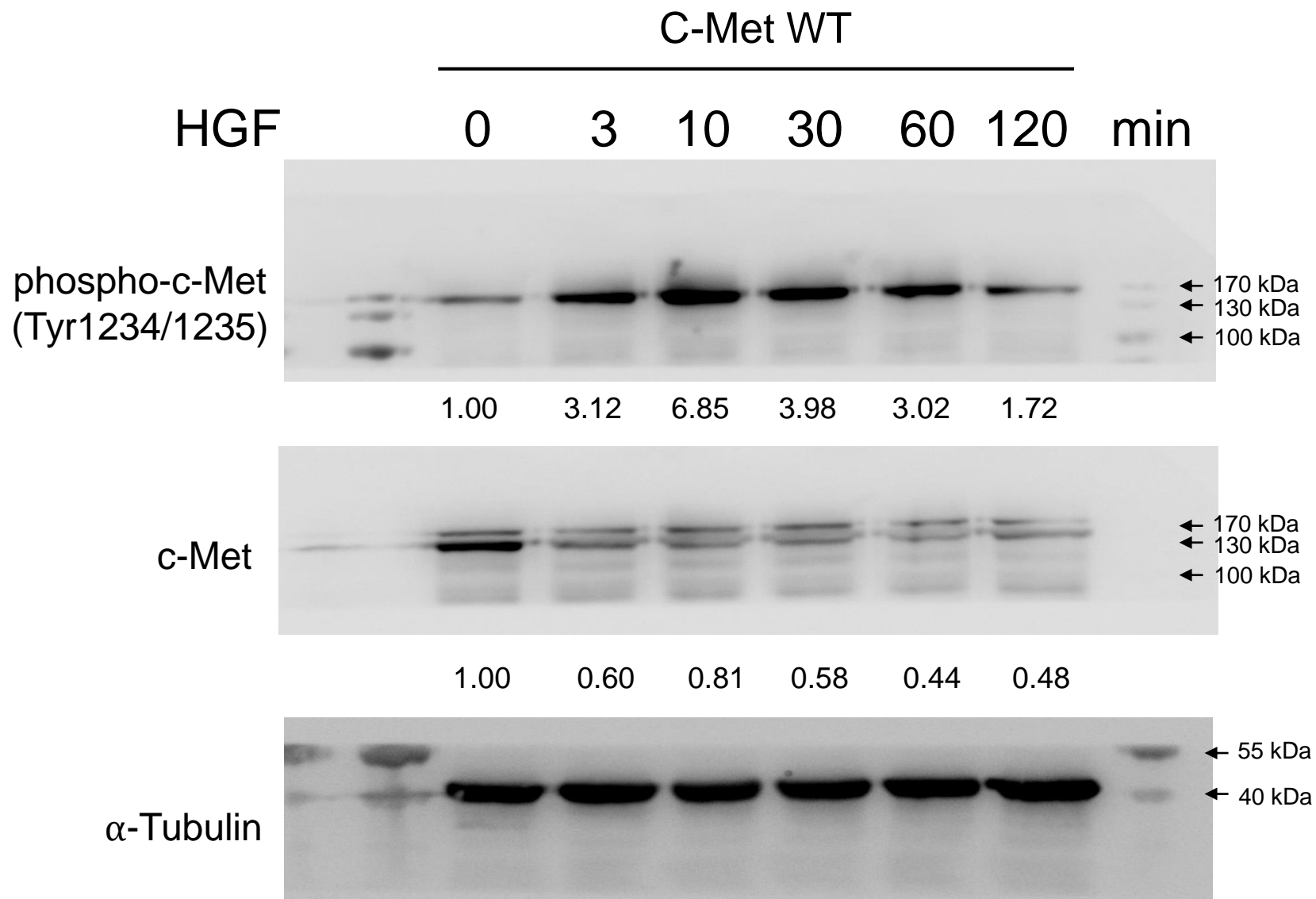

C-Met mutation  
Exon 2 (N375S)

HGF

0 3 10 30 60 120 min

phospho-c-Met  
(Tyr1234/1235)

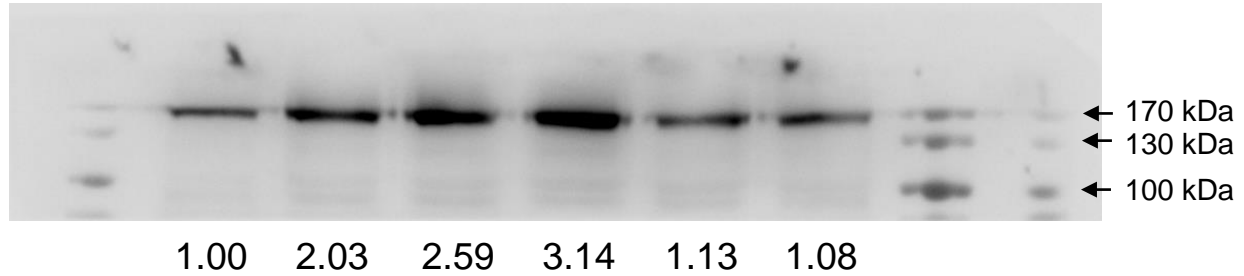

c-Met

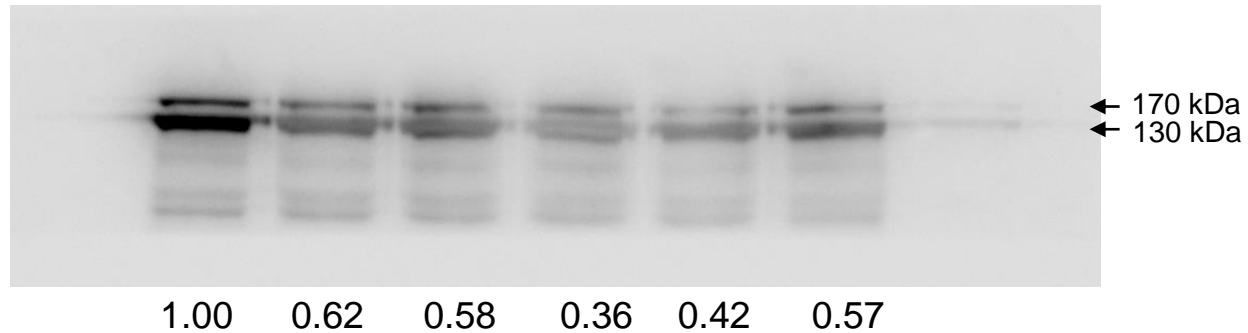

$\alpha$ -Tubulin

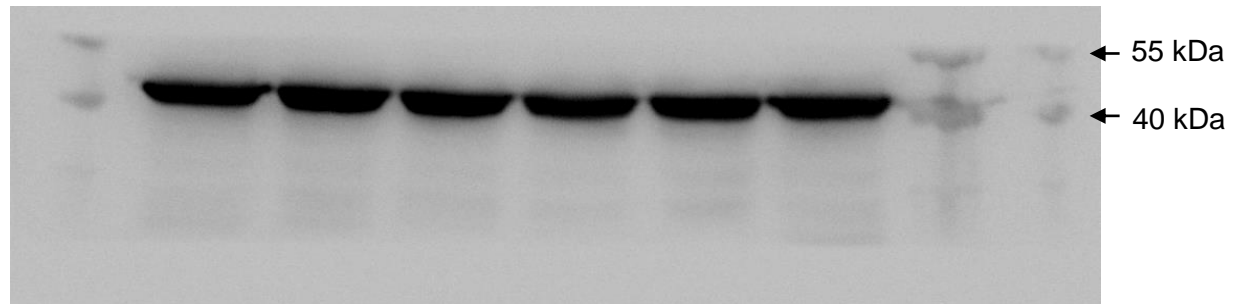

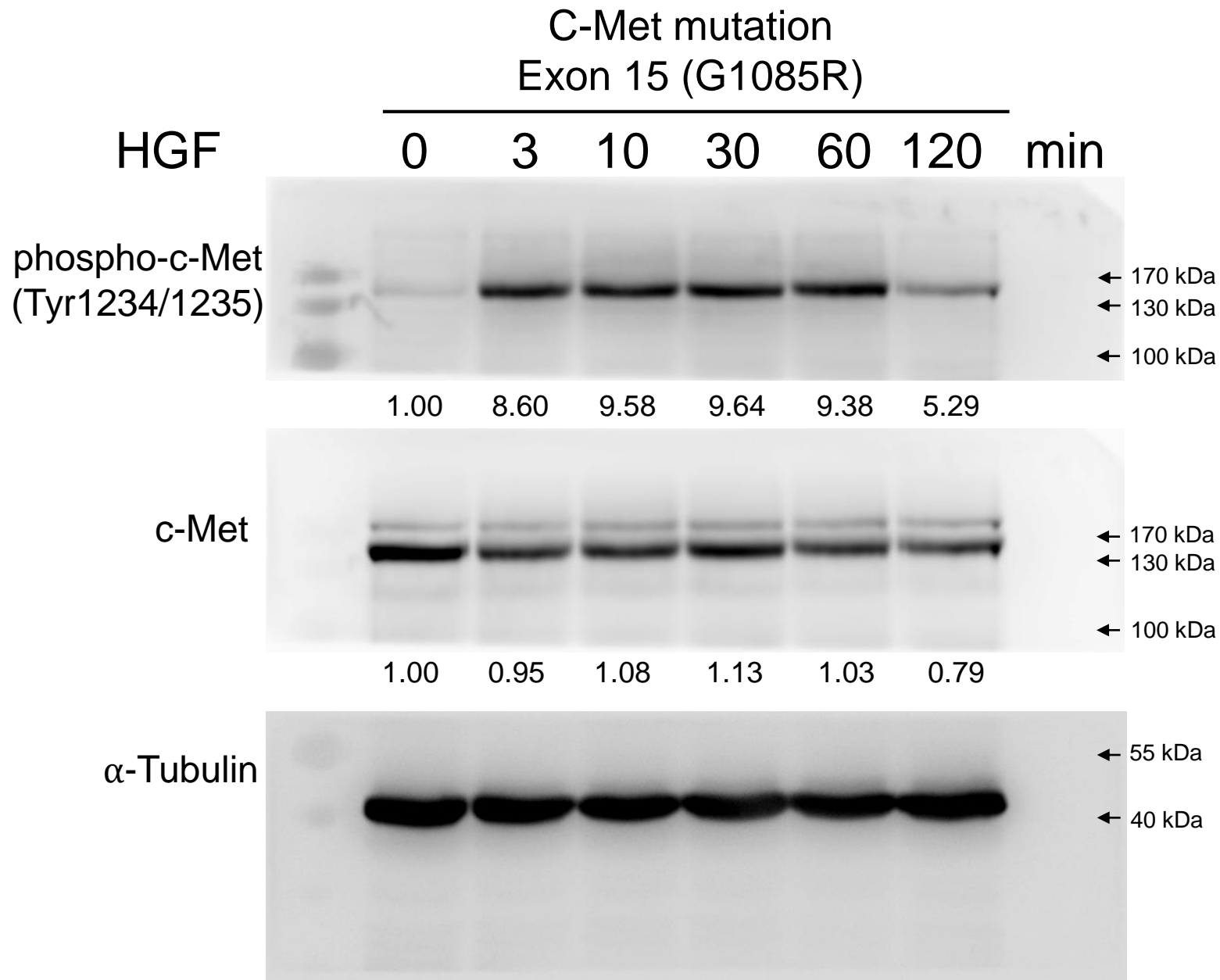

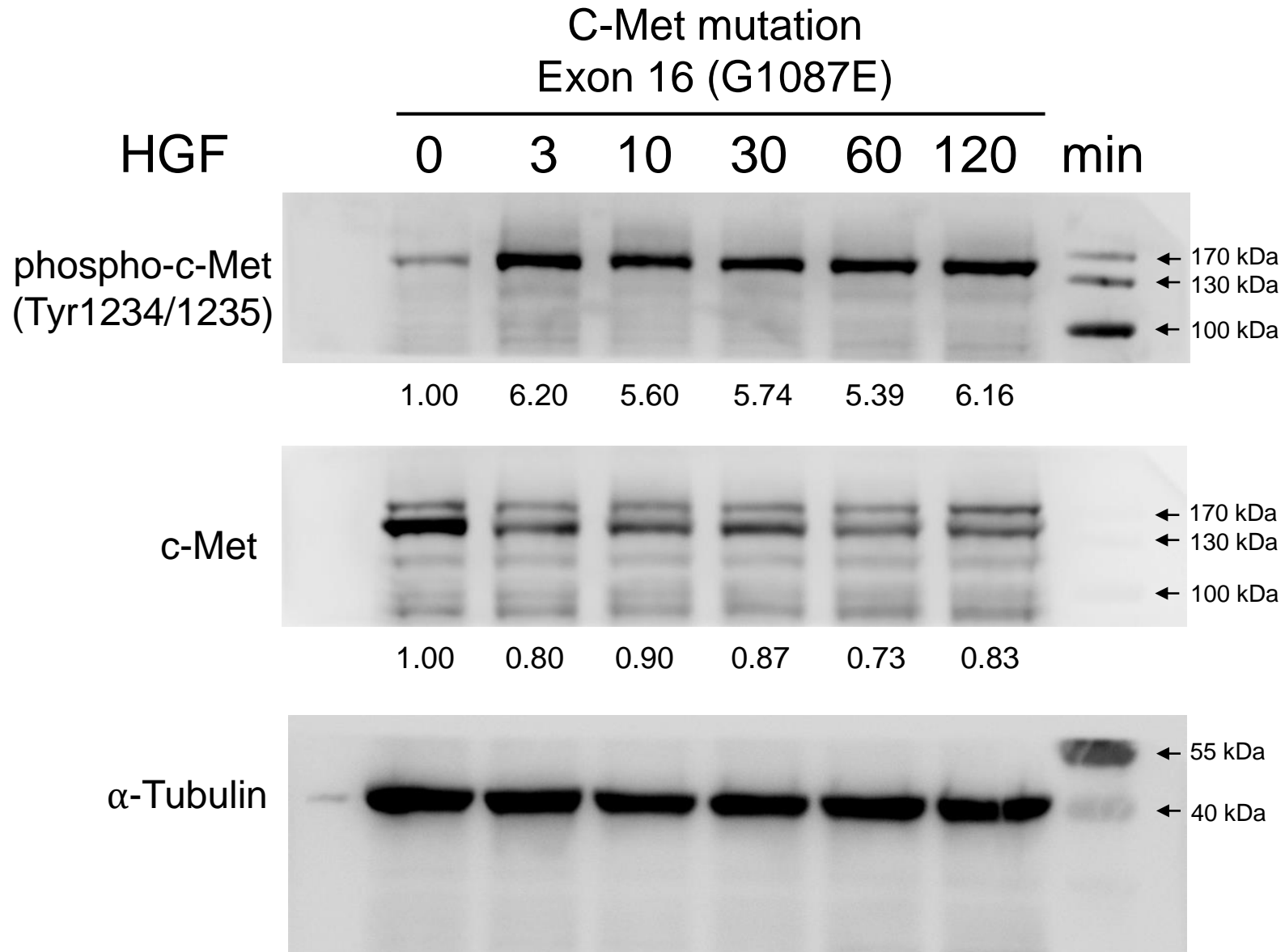

Supplement: Supplementary file 1 [file cancers-13-04231-s001.zip › cancers-1323092-supplementary-proof/supplementary/Supplementary Figure S8 c-Met WB raw data.pdf]
